# Small-Scale Variation in Fuel Loads Differentially Affects Two Co-Dominant Bunchgrasses in a Species-Rich Pine Savanna

**DOI:** 10.1371/journal.pone.0029674

**Published:** 2012-01-17

**Authors:** Paul R. Gagnon, Kyle E. Harms, William J. Platt, Heather A. Passmore, Jonathan A. Myers

**Affiliations:** 1 Department of Biological Sciences, Louisiana State University, Baton Rouge, Louisiana, United States of America; 2 Smithsonian Tropical Research Institute, Balboa, Republic of Panama; Duke University, United States of America

## Abstract

Ecological disturbances frequently control the occurrence and patterning of dominant plants in high-diversity communities like C_4_ grasslands and savannas. In such ecosystems disturbance-related processes can have important implications for species, and for whole communities when those species are dominant, yet mechanistic understanding of such processes remains fragmentary. Multiple bunchgrass species commonly co-dominate disturbance-dependent and species-rich pine savannas, where small-scale fuel heterogeneity may influence bunchgrass survival and growth following fires. We quantified how fire in locally varying fuel loads influenced dynamics of dominant C_4_ bunchgrasses in a species-rich pine savanna in southeastern Louisiana, USA. We focused on two congeneric, co-dominant species (*Schizachyrium scoparium* and *S. tenerum*) with similar growth forms, functional traits and reproductive strategies to highlight effects of fuel heterogeneity during fires. In experimental plots with either reduced or increased fuels versus controls with unmanipulated fuels, we compared: 1) bunchgrass damage and 2) mortality from fires; 3) subsequent growth and 4) flowering. Compared to controls, fire with increased fuels caused greater damage, mortality and subsequent flowering, but did not affect post-fire growth. Fire with reduced fuels had no effect on any of the four measures. The two species responded differently to fire with increased fuels – *S. scoparium* incurred measurably more damage and mortality than *S. tenerum*. Logistic regression indicated that the larger average size of *S. tenerum* tussocks made them resistant to more severe burning where fuels were increased. We speculate that locally increased fuel loading may be important in pine savannas for creating colonization sites because where fuels are light or moderate, dominant bunchgrasses persist through fires. Small-scale heterogeneity in fires, and differences in how species tolerate fire may together promote shared local dominance by different bunchgrasses.

## Introduction

Changes in populations of dominant plants can have important consequences for entire biological communities and ecosystems [1]. Plants that are dominant in terms of biomass or stature can be key contributors to ecosystem productivity (e.g. [2], [3]) and community assembly or invasibility [4]–[6]. Grime [7] asserted that to understand vegetation structure and dynamics, studies of plant dominance should have high priority because “the struggle between potential dominants provides a potent driving force for successional change and is a major determinant of the fate of subordinate species.”

Ecological perturbations can control the occurrence and patterning of potentially dominant species [8]. Such disturbances are intrinsically heterogeneous both spatially and temporally. Examples include fires [9]–[11], drought and climatic variability [12], [13], grazing [9], [14], windstorms and other gap-opening events, among others [15]–[17]. If disturbance effects are sufficiently variable at small scales, the resulting heterogeneity might enable multiple species to share dominance in the community.

Multiple C_4_ bunchgrass species frequently co-dominate the groundcover in many fire-dependent and species-rich grasslands and savannas (e.g., [9], [12], [18]–[23]). The interstices among these large, relatively fire-tolerant grasses constitute the matrix within which smaller but more diverse “subordinate species” (sensu [7]) occur. Where multiple bunchgrass species have the potential to dominate particular fire-prone sites, local heterogeneity in disturbances like fire might affect potential dominants differently [21].

Pine savannas in the southeastern USA are among the plant communities wherein multiple bunchgrass species commonly co-dominate. We use the term “savannas” to emphasize the near continuous groundcover of grasses and discontinuous pine canopy that typifies these ecosystems [24], [25]. Frequent fires regularly crop dominant bunchgrasses that account for the bulk of living herbaceous biomass in these ecosystems [18], [21]. Spatially heterogeneous fuels, especially the needles, cones and branches of overstory pines, cause local variation in fires [11], [26]–[28]. Although it has yet to be demonstrated empirically, small-scale variation in these fires is hypothesized to play a critical role in maintaining high species richness in pine savanna understories [11], [26]. A better understanding of how local variation in fires affects bunchgrass survival and growth, including how effects differ among species, should offer key insights into why species sometimes co-dominate these pine-savanna ecosystems.

We explored how variation in fire disturbance affected co-dominant perennial bunchgrasses in a species-rich pine savanna. We sought to quantify the extent to which variation in fire severity, manipulated through contrasting fuel loads, damaged and killed potentially dominant grass tussocks of different species and how the subsequent growth and reproductive output of surviving tussocks varied across species and fire treatments. In a pine savanna in southeastern Louisiana, USA, we focused on two congeneric, co-dominant bunchgrasses of similar growth form and clonal habit to highlight effects of varying fuel loads during fires. We compared fire-induced damage and mortality along with post-fire growth and flowering of bunchgrasses in experimental plots in which we either reduced or increased fuel loads compared to unmanipulated controls. Fuel loads in pine savannas vary substantially with proximity to overstory pines (e.g., [29]), and we designed our fuel treatments based on previously measured natural variability in fuel loads for this ecosystem [11]. We hypothesized that: 1) adding fuels to increase local fire severity would increase damage and mortality to the two co-dominant bunchgrasses; 2) those bunchgrass tussocks that survived fires with added fuels would grow more during the remainder of the growing season, likely owing to locally increased space and resource availability; 3) bunchgrass tussocks that survived fires with added fuels would be more likely to flower, again likely owing to locally increased space and resource availability; and 4) response of the two species to burning would differ in terms of damage, mortality and growth. Based on our results, we explored how variation in fires driven by small-scale fuel heterogeneity could be important for understanding shared plant dominance in fire-prone habitats.

## Materials and Methods

### Study site, focal organisms and experimental design

We compared effects on dominant bunchgrasses of fires burning different fuel loads at Camp Whispering Pines, a restored, species-rich pine savanna in southeastern Louisiana, USA (30° 41′ N; 90° 29′ W; mean annual temperature = 19°C, mean annual rainfall = 1626 mm [30]). The terrain is moderately dissected, 25–50 m above mean sea level. The site's Pleistocene-aged fine sands mixed with and capped by loess are among the most fertile pine savanna soils [31]. The site has never been plowed, and for 15 years had been burned biennially during the early growing season (April-May) [30] prior to the time of the experiment. The overstory is primarily longleaf pine (*Pinus palustris* Mill.) that regenerated naturally after the site was logged in the early 1900 s [24]. The pine savanna on this 270 ha site contains >300 vascular plant species (W. J. Platt *et al.*, unpublished data), including diverse groundcover forbs, shrubs and grasses at small scales (∼30 species • m^−2^; K. E. Harms et al., unpublished data). Multiple bunchgrass species are dominant in the groundcover at the site; the two most common in the focal plots were *Schizachyrium scoparium* (Michx.) Nash and *S. tenerum* Nees (see Results). For additional ecological details about the research site and its management history, see Platt et al. [30].

We manipulated fine-fuel quantities to produce small-scale heterogeneity in fires. We randomly assigned one of three levels of fine-fuel treatments to each of 24 square, 4-m^2^ plots in two separate burn units (*N* = 24×2 = 48 plots) burned under prescription on different days in the early growing season (May 10 & 14, 2007). We randomly chose plot locations away from clonal shrubs and overstory trees to reduce variability in fuel quantities (i.e., needles, cones and branches). To each of one-third of the plots in each burn unit we added 8 kg of dry, uncompacted longleaf pine needles (i.e., fuel-addition treatment), spread evenly over the plot on the same morning as the two fires. This quantity of pine straw (2 kg • m^−2^) mimicked the upper range of observed fuel loads at this relatively productive study site [11], [32]. Another third we left as control plots with unmanipulated fuels. In the remainder we clipped and removed existing biomass above 5 cm (i.e., fuel-reduction treatment). Following fuel treatments but before burning, plots contained on average 3076 g • m^−2^ (fuel addition), 1076 g • m^−2^ (control) and 444 g • m^−2^ (fuel reduction) (1SE = 57, 57 and 23 respectively) of total aboveground biomass (we estimated these quantities from nearby plots treated similarly, with fuels collected and then weighed after drying for 48 hours at 100°C). These quantities included natural herbaceous litter and any natural or added pine straw, plus such naturally occurring fine fuels as pine cones and small pine twigs. To reduce variability among plots within a given treatment, we removed any coarser woody fuels like large branches prior to burning and did not include such fuels in this estimate. During the late morning on two dry days with light breezes, we established backing fires in the two different burn units and then set head-fires that we allowed to burn through the plots. Fuel-reduction plots burned with fine-scale patchiness, while control and fuel-addition plots all burned thoroughly. Fuels in all fuel-addition plots burned almost completely to ash.

One of us (PRG) performed three separate censuses of all bunchgrasses in the interior 1-m^2^ square quadrat within each of the 48, 4-m^2^ treatment plots. For each census, he mapped and measured every tussock of grass >1-cm in basal diameter and identified each tussock to species. The first census was in late March 2007 (spring census) prior to burning. The second was in June (summer census), roughly three weeks after burning, by which time the large majority of tussocks not killed by fires were resprouting. The third census was in October/November 2007 (fall census) at the end of the growing season. Viewed from above, most tussocks were approximately elliptical in shape; measuring them consisted of 2 diameters at 90° (length and width). In addition to basal-area measurements in the fall census, he also noted whether or not each tussock was flowering (the timing of the fall census was planned to facilitate collection of these flowering data).

### Quantitative analyses

Our initial exploratory analyses focused primarily on data from the spring census (pre-fire and pre-treatment). Our dataset of tussocks included species designation and basal area from the modeled ellipses of each individual bunchgrass tussock in every quadrat. We calculated simple means for number of tussocks and basal area of each species. We also tallied the proportion of each species in every quadrat in terms of both number and basal area. Our subsequent analyses focused solely on the two common species of *Schizachyrium* (*S. scoparium* and *S. tenerum*). To evaluate statistical difference in size between *S. scoparium* and *S. tenerum* tussocks, we used log-transformed basal area (in cm^2^) of tussocks from the spring census as our response variable, species as a fixed effect and both burn unit and plot as random variables. We used a Kenward-Roger approximation to account for lack of balance in the dataset for this and all subsequent analyses (both species occurred in most but not all quadrats). We performed this analysis using the Mixed procedure in SAS v. 9.1.3 [33].

**Figure 1:**
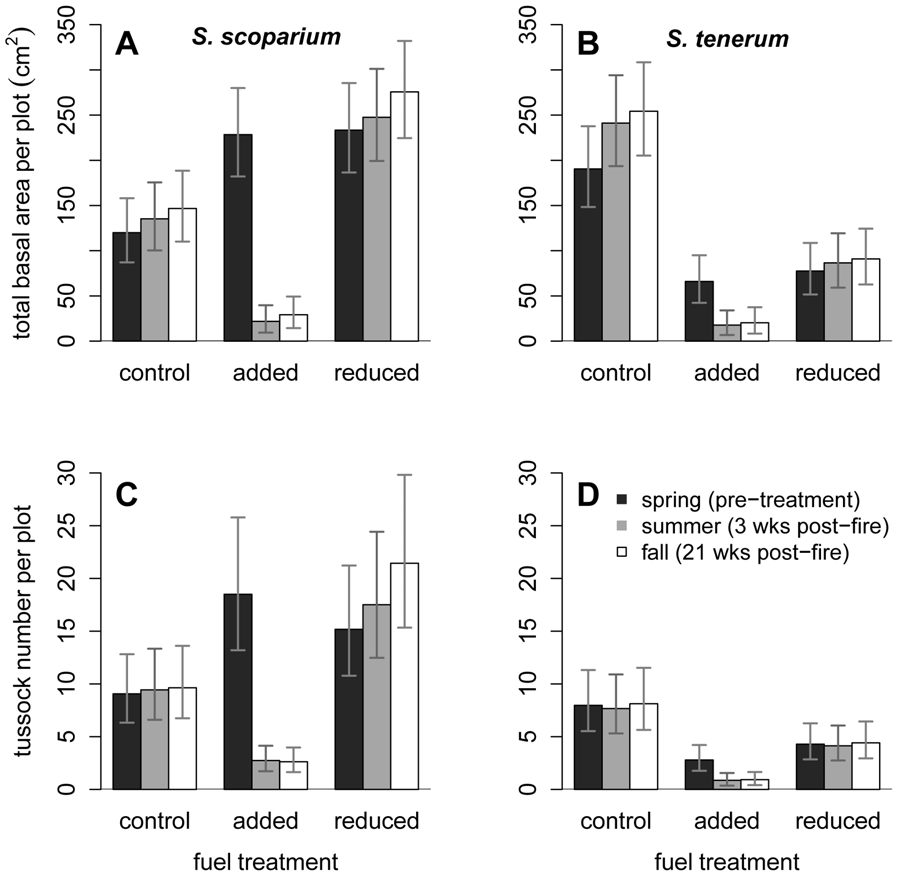
Tussock number and basal area of two *Schizachyrium* bunchgrasses in three fuel levels. Basal area (A and B) and number of tussocks (C and D) for *Schizachyrium scoparium* (A and C) and *S. tenerum* (B and D) in control, increased and reduced fuel treatments in spring, summer and fall censuses (least squares means +/−95% confidence intervals).

We examined the extent to which fires burning different fuel loads damaged *Schizachyrium* tussocks. Our response variable was total basal area of each species per plot (square-root transformed to normalize residuals). Damage was indicated by reduction in basal area per plot between the spring and summer censuses. Fuel treatment was a 3-level fixed effect applied at the whole plot level. Species functioned as a split-plot effect within fuel-treatment whole plots. The two burn units were random-effect blocks. Census was our repeated variable. Here we graphically present least-square means and standard errors from the model that included data from each of the three censuses as analogous repeated measures (Figure 1 A and B). By coincidence when we randomly assigned treatments to plots, *S. scoparium* had more individuals and total basal area in plots assigned to both fuel-added and fuel-reduced treatments, whereas *S. tenerum* had more in control plots. Because of this significant pre-treatment difference between species, in the Results we interpret tests for significance from an analogous model that treated pre-fire basal area (spring census) as a covariate and only included data from summer and fall censuses in our response variable. By thus controlling for the pre-treatment difference between species, we are able to focus our discussion on the effects of the applied experimental treatment. We ran both homogeneous and heterogeneous variance models and chose the former on the basis of lower AIC (Akaike Information Criterion; Ch. 9 in [34]). We performed this repeated measures analysis of covariance (ANCOVA) using the Mixed procedure in SAS v. 9.1.3 [33].

We next examined effects of fires burning different fuel loads on mortality of individual *Schizachyrium* tussocks. Our response variable in each plot was number of tussocks per species (log transformed to normalize residuals), with reduction in number between spring and summer censuses indicating killed tussocks. Fuel treatment was a 3-level fixed effect applied at the whole-plot level; species functioned as a split-plot effect within fuel-treatment whole plots. The two burn units were random-effect blocks. Census was our repeated variable. As per our analyses of basal area, we graphically present least-square means and standard errors from the model that considered data from each of the three censuses as analogous repeated measures (Figure 1 C and D). However, in the Results we interpret tests for significance from an analogous model that treated pre-fire number of tussocks (spring census data) as a covariate and included only data from summer and fall censuses in our response variable. We thus controlled for the pre-treatment difference between species and can more easily focus our discussion on the effects of the applied experimental treatment. We ran both homogeneous and heterogeneous variance models and chose the former on the basis of lower AIC (Ch. 9 in [34]). We performed repeated measures ANCOVA using the Mixed procedure in SAS v. 9.1.3. [33].

To separate size of tussocks from other species effects on survival, we performed logistic regression. We assigned “1” to every individual tussock tallied in our spring census that resprouted by the fall census and “0” to those that did not resprout; these survival tallies served as our binomial response variable. Fuel treatment was again our 3-level fixed effect applied at the whole-plot level, with species treated as split-plot. Basal area from the spring census served as a covariate to control for differences in initial tussock size. The two burn units were random-effect blocks. For this analysis, we used a binomial distribution with a logit link in the Glimmix procedure in SAS v. 9.1.3 [33] and simultaneously calculated odds ratios.

To determine whether survivors grew differently following fires burning different fuel loads, we analyzed individual growth of tussocks. For our response variables, we calculated growth over two time periods. The first period was the early growing season, including the first few weeks post-fire, to see how fires immediately affected tussock size (equals mean tussock basal area in summer minus spring, divided by number of tussocks in spring). The second period was for the duration of the growing season to determine whether tussocks grew different amounts where their density was reduced by fires in fuel-addition plots (equals mean basal area of tussocks in fall minus summer, divided by number of tussocks in summer). Again fuel treatment was our 3-level fixed effect applied at the whole-plot level, and species was our split-plot fixed effect. The two burn units were random-effect blocks. We ran both homogeneous and heterogeneous variance models and chose the latter (grouped by fuel treatment) based on lowest AIC (Ch. 9 in [34]). We again used the Mixed procedure in SAS v. 9.1.3 for this analysis [33].

To determine whether fires burning different fuel loads affected subsequent flowering by surviving tussocks, we again used logistic regression. To avoid problems of unbalanced data in this analysis, we focused on *S. scoparium* because *S. tenerum* was not present in several plots. We assigned “1” to every flowering tussock of *S. scoparium* in our fall census and “0” to non-flowering tussocks; resulting tallies served as our binomial response variable. Fuel treatment was our 3-level fixed effect. Basal area from the fall census served as a covariate to control for differences in tussock sizes. The two burn units were random-effect blocks. For this analysis, we used a binomial distribution with a logit link in the Glimmix procedure in SAS v. 9.1.3 [33] and simultaneously calculated odds ratios.

## Results

Within study plots, the two *Schizachyrium* species were dominant among all bunchgrass species in terms of both basal area and number. In our spring census, they accounted for 92% of total bunchgrass basal area and 91% of all individual tussocks. Of the two, *S. scoparium* accounted for 51% of total basal area and 63% of total number, whereas *S. tenerum* accounted for 41% and 28%, respectively. This disparity indicates that on average, *S. scoparium* tussocks were more numerous but smaller than those of *S. tenerum* (back-transformed lsmeans = 7.26 vs. 12.94 cm^2^ {+/−1SE = 5.87, 8.94 vs. 10.52, 15.87}, respectively; *F*_1,554_ = 40.20, *P*<0.001). Within our two burn units, several species in the genus *Andropogon* combined to account for 5% of total basal area and 6% of individual tussocks, whereas the genus *Aristida* (all *A. purpurea* Nutt.) accounted for 1% of each. Subsequent analyses focus exclusively on the two co-dominant *Schizachyriums*.

As expected, fire with added fuels caused substantially more damage to tussocks than fire with control or reduced fuel levels (Figure 1A and B). Repeated measures ANCOVA using basal area in summer and fall as the response variable and basal area in spring (pre-fire) as a pre-treatment covariate indicated a significant effect of fuel treatment (*F*_2,69.3_ = 10.37, *P*<0.001); where fuels were added, there was significantly less bunchgrass basal area after fires than with either control or reduced fuels, which were similar. There was also a significant effect of census (*F*_1,81_ = 22.42, *P*<0.001); mean total basal area in the fall increased significantly over summer levels, reflecting the fact that tussocks increased in size during the growing season. Although there was no main effect of species (*F*_1,58.2_ = 0.02, *P* = 0.887), species did interact significantly with fuel treatments (*F*_2,58_ = 3.61, *P* = 0.033). This interaction was caused by a marked difference between the two species where we added fuels; whereas these fires reduced basal area of *S. tenerum* by approximately half (51.8%), this same treatment reduced basal area of *S. scoparium* by 84.2%. There was no effect on basal area of burning with either reduced or control fuels; for both species in these two treatments, basal area slightly increased (non-significantly) over pre-fire levels. Pre-fire basal area was predictive of post-fire basal area (*F*_1,62_ = 268.56, *P*<0.001), as was the interaction of the covariate with fuel treatment (*F*_2,65.4_ = 11.25, *P*<0.001) and the three-way interaction of pre-fire basal area with fuel and species (*F*_3,70.2_ = 5.56, *P* = 0.002). No other covariate interactions were statistically significant, so we excluded those from our analysis (as per [34]; see Table S1).

Fire with added fuels also caused substantially more mortality to tussocks than fire with either control or reduced fuel levels (Figure 1C and D). ANCOVA using number of individual tussocks in the summer and fall as the response variable and number in springtime as a pre-treatment covariate indicated a significant effect of fuel treatment (*F*_2,43.9_ = 34.35, *P*<0.0001); where fuels were added, there were significantly fewer tussocks after fires than with either control or reduced fuels, which were similar. Effect of census was also significant (*F*_1,90_ = 9.14, *P* = 0.003); mean number of combined tussocks in the fall increased significantly over summer levels, reflecting establishment of new recruits during the growing season. There was no main effect of species (*F*_1,48.8_ = 1.38, *P* = 0.245), but as with basal area (above), a species-by-fuel treatment interaction was significant (*F*_2,44.7_ = 6.50, *P* = 0.003). Whereas fire with added fuels killed 2/3 of *S. tenerum* tussocks (67.1%), this treatment killed 4/5 of *S. scoparium* individuals (80.5%). By contrast, fire with neither reduced nor control fuel levels caused any detectable mortality. In these latter two fuel treatments, and especially in reduced fuel plots, the number of *S. scoparium* individuals increased during the 3 censuses, whereas the number of *S. tenerum* individuals remained constant, as indicated by a significant 3-way interaction of fuel treatment, census and species (*F*_2,90_ = 3.32, *P* = 0.041). Pre-fire number of tussocks was predictive of post-fire numbers (*F*_1,78_ = 83.2, *P*<0.001), but no covariate interactions were significant (see Table S2).

Larger tussocks of both species were significantly more likely to survive fires. To untangle the effect of tussock size from other possible species effects, we used logistic regression to analyze tussock survival as a binomial response variable and initial tussock size as a covariate. The effect of fuel treatment was significant (*F*_2,68.2_ = 59.00, *P*<0.001) and initial tussock size predicted tussock fate (*F*_1,1256_ = 17.00, *P*<0.001). With tussock size accounted for, there was no additional difference in survival between the two *Schizachyrium* species (*F*_1,1256_ = 0.14, *P* = 0.7114), nor were any higher order interactions significant (see Table S3). According to the odds ratios, tussock survival was not statistically different between fires with control vs. reduced fuel levels (odds ratio = 1.776, 95% confidence = 0.740, 4.262; note that this odds-ratio indicates {non-significantly} higher survival rates in control than in reduced fuels). However, the odds of tussock survival were significantly lower in fires with added fuels than in the other two treatments (for added-fuels vs. removed-fuels, odds ratio = 0.021, 95% confidence = 0.010, 0.044). From this analysis we conclude that the difference in survival between the two *Schyzachyrium* species in fires with added fuels was mainly a function of the larger average size of *S. tenerum* tussocks.

Fires with added fuels caused substantial reduction in tussock size, but growth in basal area after the fires was similar across fuel treatments. Our measure of tussock growth was mean growth per tussock (in cm^2^) at the plot level. Comparing spring (pre-fire) to summer (immediate post-fire) census data, the effect of fuel treatment was again significant (*F*_2,48.6_ = 30.87, *P*<0.001) but there was no effect of species nor fuel-treatment-by-species interaction (*F*_1,63.5_ = 1.59, *P* = 0.212 and *F*_2,48.6_ = 1.95, *P* = 0.514, respectively). Growth in the post-fire environment (tussock size in the fall versus summer census) indicated no effect of fuel-treatment (*F*_2,35.1_ = 0.84, *P* = 0.441), species (*F*_1,21.5_ = 0.62, *P* = 0.438) nor their interaction (*F*_2,19.4_ = 0.11, *P* = 0.896; see Table S4).

Tussocks that survived fires with added fuels were more likely to flower than tussocks growing where fuels were removed. Only *S. scoparium* had multiple individuals present in almost all plots, so our flowering analysis focused on that species to ensure adequate replication. We modeled likelihood of flowering as a binomial response variable using logistic regression, with individual tussock size (i.e., basal area) at census 3 as a covariate. As expected, tussock size was highly predictive of the likelihood of flowering (*F*_1,572_ = 42.67, *P* = 0.001); larger tussocks were more likely to flower. The overall effect of fuel-treatment was marginally significant (*F*_2,30.77_ = 3.09, *P* = 0.060; see Table S5). Odds of flowering for *S. scoparium* tussocks were similar for control and reduced plots (odds ratio = 1.492, 95% confidence = 0.700, 3.183) and between added-fuels and controls. However, surviving *S. scoparium* tussocks had increased odds of flowering in added-fuels than in reduced-fuels (odds ratio = 3.244, 95% confidence = 1.241, 8.479).

## Discussion

### Fire with added fuels alters bunchgrass survivorship, growth and fecundity

Small-scale variation in fuels and resulting fire heterogeneity can have important effects on the survivorship, growth and fecundity of dominant species in fire-prone habitats. The two *Schizachyrium* species were much more prevalent in our research plots than all other bunchgrasses combined. Together, *S. scoparium* and *S. tenerum* composed 91% of all identifiable tussocks and 92% of total bunchgrass basal area. Fires with added fuels significantly increased mortality and reduced basal area (i.e., caused negative growth) of these two co-dominants, and these effects persisted through the end of the growing season. The result was that our fire with added fuels treatment opened substantial new space. Reduced bunchgrass dominance could result if other species are able to exploit and monopolize this new space. Although the detrimental effect on bunchgrasses of adding fuels was to be expected, virtually every tussock in both control and reduced fuel treatments survived burning with zero reduction in basal area. Some threshold of fuel loading appeared to be operating, below which these bunchgrasses were impervious to fire; clearly the naturally occurring fuel loads in our unmanipulated controls were below this threshold. That reduced fuels and concordant reduction in fire intensity offered no benefit to bunchgrass tussocks compared to controls suggests that both focal species were well adapted to fires like those in our control plots.

Longleaf pines are important drivers of potential dynamism in the understory through their production of fuels. Historically, longleaf pine trees occurred patchily on the landscape [24], [25], [35]; the distribution of their fuels would have been similarly patchy, even where their fuels were sufficiently continuous to carry fire throughout [26], [27], [29]. The substantial quantities of needles, cones and branches dropped by pines on the understory generate local hotpots during frequent fires [25], [29], [36], [37]. These fuels vary temporally as well as spatially, as when hurricanes and other intense disturbances generate treefalls that drop fuels in irregular pulses. Our fuel addition treatment was designed to mimic the heavy loads of fine and coarse fuels under treefalls or beneath standing pines after several years without fire; had we included plots directly under trees, fuel quantities in control plots would have been substantially greater. Fire is clearly a potent ecological filter [38]–[40]; our results indicate that in habitats like this one, the strength of this filtering may vary substantially with the location of individual plants and perhaps even single branches of, in this case, pine trees. Our results highlight the importance of fuel heterogeneity in pine savannas for locally reducing bunchgrass basal area and altering local survivorship of dominant bunchgrasses. If bunchgrasses compete with other species for limited resources, the resulting opening of new space at small scales may facilitate colonization by other species and thus support the maintenance of high diversity in this species-rich ecosystem [32]. Alternatively, if bunchgrasses facilitate the establishment of non-dominant species (as suggested by [41]), then this new open space might remain so until ruderals or other colonizing species are able to take hold.

After the fires, growth of individual tussocks showed no indication of density dependence, but rate of flowering increased following more severe burning. Because of fire-induced mortality, plants that survived fire with added fuels occurred at substantially lower densities than plants in our control and reduced-fuels treatments. We expected basal area of these sparsely growing plants to increase faster than that of their counterparts growing at higher densities, but this was not the case. Our fuel treatment affected basal area only during the brief interval that included burning, and during that interval, plant basal area decreased only where we added fuels; fuel treatment had no effect on post-fire growth rates. Unlike post-fire growth, differential rates of flowering by *S. scoparium* did suggest possible density dependent influences. The more widely separated plants that survived fire with added fuels had higher odds of flowering than more densely growing counterparts where fuels were reduced. These results suggest that for these bunchgrasses growing at such densities, crowding has an effect on reproduction but not growth. Alternatively, this increase in flowering could have been a compensatory stress response in plants that were more severely damaged in the added fuel plots. Growth of surviving tussocks did not accelerate after their density decreased, suggesting that the new space created by burning was likely to persist for some time, and so remain available for colonization by other species where space is limiting.

### Effects of added fuels differed between the two co-dominants

Our study of two co-dominant bunchgrasses is unique in that we explore implications for species of within-fire heterogeneity at the neighborhood level driven by variation in fuel loading. Although many studies have explored the effects of fire on dominant bunchgrasses, these have typically viewed fire as a uniform disturbance at the landscape scale. None that we are aware of have focused on the ecological implications of species differences in the context of small-scale variation in individual fires.

The effect of fire with added fuels was measurably different on the two dominant bunchgrass congeners both in terms of damage and mortality. Tussocks of *S. scoparium* suffered proportionally greater mortality and loss of basal area in fires with added fuels than tussocks of *S. tenerum*. This outcome suggests that *S. tenerum* should be relatively more frequent where fuel loads are elevated, such as under pine trees and treefalls, whereas *S. scoparium* should be relatively more frequent in open areas away from large trees in old-growth pine savannas [24], [35]. Indeed, we have observed elsewhere at this study site that the relative abundance of *S. tenerum* was increased under pines compared to in gaps (William J. Platt, unpublished data). Others have demonstrated effects of fires in increased fuels under pines that exclude potential woody competitors [11], [29], [35], [37]; our results indicate that locally heavy fuel loads favor some closely related herbaceous species over others in the same functional group.

**Figure 2:**
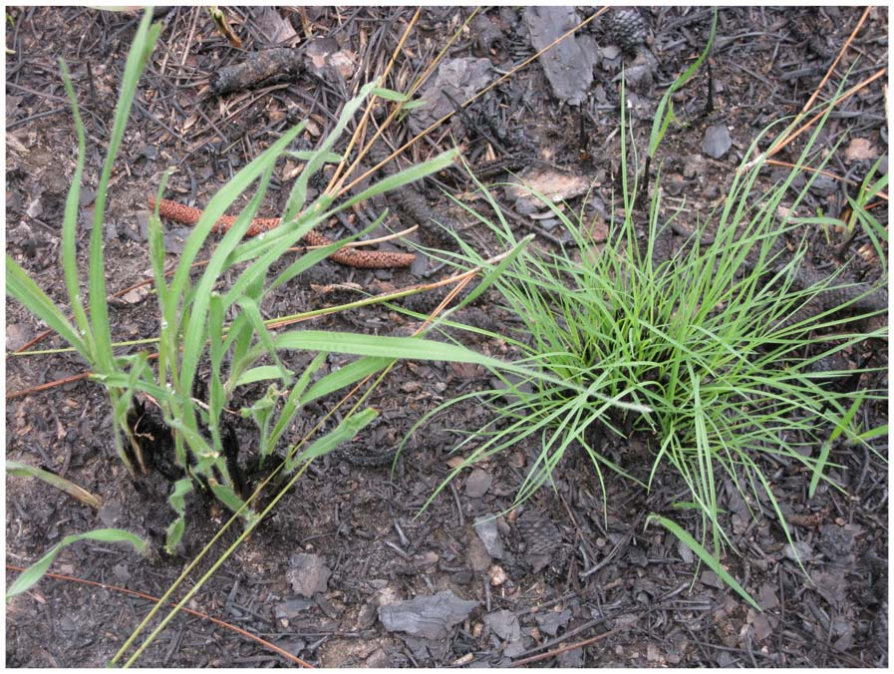
Side-by-side view of the co-dominant bunchgrass congeners. The two species, *Schizachyrium scoparium* (left) and *S. tenerum* (right), here are both resprouting after a recent prescribed fire.

Tussock size was the main determinant of both mortality and degree of damage in fires with added fuels. Larger tussocks were more likely to survive and suffered proportionally less damage. Tussocks of *S. tenerum* were larger on average than those of *S. scoparium*, and in our analyses, this key difference explained the lower rates of damage and mortality of *S. tenerum*. We observed that at our field site in southeastern Louisiana, tussocks of *S. tenerum* were more circular in shape and more densely packed with thinner, finer culms (Figure 2). Culms of *S. scoparium* were coarser and less densely packed in tussocks, which were often irregularly (amoeboid) shaped. Our results indicate that beyond basal area, such morphological differences were inconsequential in predicting tussock ability to endure hotter fires, although additional investigations of bunchgrass morphology and fire effects are merited.

Our findings suggest how heterogeneity of fuels might differentially affect population dynamics of dominant bunchgrass species in pine savannas. Fire with added fuels in our study reduced survivorship and caused negative growth (our measure of damage equates to negative growth), and plants exhibited no compensatory growth after fires. These negative impacts from fire with added fuels were more pronounced for *S. scoparium* than for its co-dominant congener. Numerous demographic studies have concluded that for long-lived iteroparous plants like perennial bunchgrasses, rates of growth and especially adult survivorship consistently influence population growth rates (λ) much more than do other vital rates [42]–[48]. Given this, we speculate that these differential effects will be reflected in both species' population growth rates beyond this one important year during which these plants burned. Our findings indicate that within given fires, local pockets of high severity can favor one co-dominant over another; thus, small-scale heterogeneity in fires (especially as a function of the location of pine trees), and differences in how species tolerate those fires may combine to promote shared local dominance by multiple bunchgrass species.

## Supporting Information

Table S1 Results of repeated measures ANCOVA of bunchgrass basal area.
(DOCX)

Table S2 Results of repeated measures ANCOVA of tussock number.
(DOCX)

Table S3 Results of logistic regression analysis of tussock survival.
(DOCX)

Table S4 Results of ANOVA of bunchgrass growth.
(DOCX)

Table S5 Results of logistic regression analysis of flowering in *S. scoparium*.
(DOCX)
