# Supplementary material for: Small-Scale Variation in Fuel Loads Differentially Affects Two Co-Dominant Bunchgrasses in a Species-Rich Pine Savanna
Source: PLoS One. 2012 Jan 17;7(1):e29674. doi: 10.1371/journal.pone.0029674 (PMC3260174; doi:10.1371/journal.pone.0029674)
Supplement: Table S1 — Results of repeated measures ANCOVA of bunchgrass basal area. (DOCX) [file pone.0029674.s001.docx]

**Table S1: Results of repeated measures ANCOVA of bunchgrass basal area**

| Source of Variation: | NDF | DDF | F | *P* |
| --- | --- | --- | --- | --- |
| Repeated measures fixed effects on basal area |  |  |  |  |
| Fuel | 2 | 69.3 | 10.37 | <0.001 |
| Species | 1 | 58.2 | 0.02 | 0.887 |
| Census | 1 | 81 | 22.42 | <0.001 |
| Fuel x Species | 2 | 58 | 3.61 | 0.033 |
| Fuel x Census | 2 | 81 | 0.12 | 0.888 |
| Species x Census | 1 | 81 | 1.87 | 0.176 |
| Fuel x Species x Census | 2 | 81 | 0.51 | 0.600 |
| Pre-treatment basal area (covariate) | 1 | 62 | 268.56 | <0.001 |
| Pre-treatment basal area x Fuel | 2 | 65.4 | 11.25 | <0.001 |
| Pre-treatment basal area x Fuel x Species | 3 | 70.2 | 5.56 | 0.002 |
|  |  |  |  |  |

Basal area at the time of the spring census treated as a pre-treatment covariate. NDF = numerator degrees of freedom; DDF = denominator degrees of freedom based on Kenward-Roger approximation.
