# Supplementary material for: Small-Scale Variation in Fuel Loads Differentially Affects Two Co-Dominant Bunchgrasses in a Species-Rich Pine Savanna
Source: PLoS One. 2012 Jan 17;7(1):e29674. doi: 10.1371/journal.pone.0029674 (PMC3260174; doi:10.1371/journal.pone.0029674)
Supplement: Table S2 — Results of repeated measures ANCOVA of tussock number. (DOCX) [file pone.0029674.s002.docx]

**Table S2: Results of repeated measures ANCOVA of tussock number**

| Source of Variation: | NDF | DDF | F | *P* |
| --- | --- | --- | --- | --- |
| Repeated measures fixed effects on tussock number |  |  |  |  |
| Fuel | 2 | 43.9 | 34.35 | <0.001 |
| Species | 1 | 48.8 | 1.38 | 0.245 |
| Census | 1 | 90 | 9.14 | 0.003 |
| Fuel x Species | 2 | 44.7 | 6.50 | 0.003 |
| Fuel x Census | 2 | 90 | 4.15 | 0.019 |
| Species x Census | 1 | 90 | 0.13 | 0.719 |
| Fuel x Species x Census | 2 | 90 | 3.32 | 0.041 |
| Pre-treatment tussock number (covariate) | 1 | 78 | 83.20 | <0.001 |
|  |  |  |  |  |

Number at the time of the spring census treated as a pre-treatment covariate. NDF = numerator degrees of freedom; DDF = denominator degrees of freedom based on Kenward-Roger approximation.
