# Supplementary material for: Small-Scale Variation in Fuel Loads Differentially Affects Two Co-Dominant Bunchgrasses in a Species-Rich Pine Savanna
Source: PLoS One. 2012 Jan 17;7(1):e29674. doi: 10.1371/journal.pone.0029674 (PMC3260174; doi:10.1371/journal.pone.0029674)
Supplement: Table S3 — Results of logistic regression analysis of tussock survival. (DOCX) [file pone.0029674.s003.docx]

**Table S3: Results of logistic regression analysis of tussock survival**

| Source of Variation: | NDF | DDF | F | *P* |
| --- | --- | --- | --- | --- |
| Repeated measures fixed effects on tussock number |  |  |  |  |
| Basal area | 1 | 1256 | 17.00 | <0.001 |
| Fuel treatment | 2 | 68.2 | 59.00 | <0.001 |
| Species | 1 | 1256 | 0.14 | 0.711 |
| Basal area x Fuel treatment | 2 | 1256 | 1.94 | 0.143 |
| Basal Area x Species | 1 | 1256 | 0.85 | 0.357 |
| Fuel Treatment x Species | 2 | 1256 | 0.84 | 0.430 |
| Basal Area x Fuel Treatment x Species | 2 | 1256 | 0.40 | 0.671 |
|  |  |  |  |  |

Basal area, fuel treatment and species included as fixed effects. NDF = numerator degrees of freedom; DDF = denominator degrees of freedom based on Kenward-Roger approximation.
