# Supplementary material for: Small-Scale Variation in Fuel Loads Differentially Affects Two Co-Dominant Bunchgrasses in a Species-Rich Pine Savanna
Source: PLoS One. 2012 Jan 17;7(1):e29674. doi: 10.1371/journal.pone.0029674 (PMC3260174; doi:10.1371/journal.pone.0029674)
Supplement: Table S4 — Results of ANOVA of bunchgrass growth. (DOCX) [file pone.0029674.s004.docx]

**Table S4: Results of ANOVA of bunchgrass growth**

| Source of Variation: | NDF | DDF | F | *P* |
| --- | --- | --- | --- | --- |
| Change in tussock basal area from spring to summer |  |  |  |  |
| Fuel treatment | 2 | 48.6 | 30.87 | <0.001 |
| Species | 1 | 63.5 | 1.59 | 0.212 |
| Fuel treatment x Species | 2 | 48.6 | 1.95 | 0.154 |
|  |  |  |  |  |
| Change in tussock basal area from summer to fall |  |  |  |  |
| Fuel treatment | 2 | 35.1 | 0.84 | 0.441 |
| Species | 1 | 21.5 | 0.62 | 0.438 |
| Fuel treatment x Species | 2 | 19.4 | 0.11 | 0.896 |
|  |  |  |  |  |

Mean tussock growth/shrinkage per plot between spring and summer censuses, and summer and fall censuses. Growth/shrinkage calculated as (mean basal area per plot at time t+1 minus mean basal area at time t), divided by number of tussocks at time t, yielding “mean growth per tussock” in cm^2^. Fuel treatment and species are included as fixed effects. NDF = numerator degrees of freedom; DDF = denominator degrees of freedom based on Kenward-Roger approximation.
