# Supplementary material for: Small-Scale Variation in Fuel Loads Differentially Affects Two Co-Dominant Bunchgrasses in a Species-Rich Pine Savanna
Source: PLoS One. 2012 Jan 17;7(1):e29674. doi: 10.1371/journal.pone.0029674 (PMC3260174; doi:10.1371/journal.pone.0029674)
Supplement: Table S5 — Results of logistic regression analysis of flowering in S. scoparium. (DOCX) [file pone.0029674.s005.docx]

**Table S5: Logistic regression analysis of flowering in *S. scoparium***

| Source of Variation: | NDF | DDF | F | *P* |
| --- | --- | --- | --- | --- |
| Logistic regression of *S. scoparium* post-fire flowering |  |  |  |  |
| Fuel treatment | 2 | 30.77 | 3.09 | 0.060 |
| Basal area | 1 | 572 | 42.67 | <0.001 |
|  |  |  |  |  |

Fuel treatment and tussock basal area included as fixed effects. NDF = numerator degrees of freedom; DDF = denominator degrees of freedom based on Kenward-Roger approximation.
